# Agricultural intensification reduces microbial network complexity and the abundance of keystone taxa in roots

**DOI:** 10.1038/s41396-019-0383-2

**Published:** 2019-03-08

**Authors:** Samiran Banerjee, Florian Walder, Lucie Büchi, Marcel Meyer, Alain Y. Held, Andreas Gattinger, Thomas Keller, Raphael Charles, Marcel G. A. van der Heijden

**Affiliations:** 1 Agroscope, Department of Agroecology & Environment, Reckenholzstrasse 191, 8046 Zürich, Switzerland; 2 Agroscope, Plant Production Systems, Route de Duillier 50, 1260 Nyon, Switzerland; 3 Natural Resources Institute, University of Greenwich, London, UK; 4 Research Institute of Organic Agriculture FiBL, 5070 Frick, Switzerland; 5 Justus-Liebig University Giessen, Organic Farming with focus on Sustainable Soil Use, Karl-Glöckner-Str. 21C, 35394 Giessen, Germany; 6 Swedish University of Agricultural Sciences, Department of Soil & Environment, Box 7014, 75007 Uppsala, Sweden; 7 Research Institute of Organic Agriculture FiBL, Jordils 3, 1001 Lausanne, Switzerland; 8 Department of Plant and Microbial Biology, University of Zürich, 8008 Zürich, Switzerland

**Keywords:** Microbial ecology, Ecology

## Abstract

Root-associated microbes play a key role in plant performance and productivity, making them important players in agroecosystems. So far, very few studies have assessed the impact of different farming systems on the root microbiota and it is still unclear whether agricultural intensification influences the structure and complexity of microbial communities. We investigated the impact of conventional, no-till, and organic farming on wheat root fungal communities using *PacBio SMRT sequencing* on samples collected from 60 farmlands in Switzerland. Organic farming harbored a much more complex fungal network with significantly higher connectivity than conventional and no-till farming systems. The abundance of keystone taxa was the highest under organic farming where agricultural intensification was the lowest. We also found a strong negative association (*R*^2^ = 0.366; *P* < 0.0001) between agricultural intensification and root fungal network connectivity. The occurrence of keystone taxa was best explained by soil phosphorus levels, bulk density, pH, and mycorrhizal colonization. The majority of keystone taxa are known to form arbuscular mycorrhizal associations with plants and belong to the orders *Glomerales*, *Paraglomerales*, and *Diversisporales*. Supporting this, the abundance of mycorrhizal fungi in roots and soils was also significantly higher under organic farming. To our knowledge, this is the first study to report mycorrhizal keystone taxa for agroecosystems, and we demonstrate that agricultural intensification reduces network complexity and the abundance of keystone taxa in the root microbiome.

## Introduction

Agricultural intensification is one of the most pervasive problems of the twenty-first century [1]. To keep pace with the ever-increasing human population, the total area of cultivated land worldwide has increased over 500% in the last five decades [2] with a 700% increase in the fertilizer use and a several-fold increase in pesticide use [3, 4]. Agricultural intensification has raised a wide range of environmental concerns, including poor nutrient-use efficiency, enhanced greenhouse gas emissions, groundwater eutrophication, degradation of soil quality, and soil erosion [4, 5]. Alternate farming systems such as conservation agriculture (e.g., no-till) and organic farming have been widely adopted to reduce such adverse environmental effects [6–8]. Organic arable lands represent 2.5% of the total arable lands in Europe, and over 3.5% in Switzerland [9]. The adoption of no-till globally has increased by ~233% in the last decade and it is over 3% of the total arable lands in Switzerland [10]. These farming systems are adopted to maintain environmental sustainability and ecosystems services, and at the heart of ecosystem services lies the contribution of microbial communities [11–13].

Microbial communities play an indispensable role in ecosystems and render a wide range of services [12, 14–16]. In agroecosystems, microbes modulate a number of processes, including nutrient cycling, organic matter decomposition, soil aggregate stabilization, symbiotic and pathogenic interactions with plants, and thereby play an essential role in the productivity and sustainability of agroecosystems [5, 12, 17]. The agricultural intensification with high resource use and low crop diversity can affect soil- and plant-associated microbiota, with subsequent impact on ecosystem services [18, 19]. Increasing adoption of no-till and organic farming also warrants an investigation of their effects on microbial communities. Previous studies comparing the effects of conventional, no-till, and organic farming have mostly focused on the soil microbiome [6, 8, 20–22], and our understanding of the impact of these farming systems on root-associated microbiota is minimal.

Root-associated microbiota plays a key role in determining the above-ground productivity [23–26]. No-till farming may affect root architecture and root distribution in soil, with a subsequent effect on microbial recruitment into the roots [27]. However, very few studies have assessed the effect of no-tillage on root microbial communities, and the ones that investigated root microbiota have only focused on root bacteria [28] or specific fungal groups, including arbuscular mycorrhizal fungi (AMF) using traditional techniques [29, 30]. Furthermore, the impact of agricultural intensification on the overall root fungal communities is still poorly understood [31, 32]. Plant root harbors a diverse assemblage of endophytic fungi that form symbiotic, parasitic, or pathogenic associations, and through such associations, play a key role in plant diversity, community composition, and performance [26, 33, 34]. The widespread symbiosis of AMF and the array of benefits rendered by these fungi are now well established [35, 36]. Moreover, mycorrhiza like endophytes, *Piriformospra indica*, also promote plant growth, stress tolerance and induce local and systemic resistance to pathogens [37]. *Trichoderma* spp. have also been shown to grow endophytically and enhance plant growth and systemic resistance to plant pathogens [38]. Thus, the structure and composition of root fungal communities play an important role in agroecosystems, and yet the effect of agricultural intensification on root fungal communities remains poorly understood.

The structure of a microbiome has substantial effects on its functioning [39]. However, studying the structure of a microbiome is not simple mainly due to complex interrelationships among the myriad of members. Microbial co-occurrence networks can unravel such relationships and offer insight into community structure [40–43]. Network analysis has been found particularly useful in recent years to understand how microbe–microbe associations change in response to environmental parameters [42, 44–47]. Network scores can also be used to statistically identify the keystone taxa, i.e., taxa that have a large influence in the community [34, 48, 49]. A recent study has shown that despite being numerically inconspicuous, keystone taxa confer greater biotic connectivity to the community and thus can be indicators of community shifts and compositional turnover [50]. It has also been observed that the impact of abiotic factors and host genotypes on the plant microbiome is facilitated via keystone taxa [51], and the root microbial network complexity is linked to plant survival [52]. Agricultural intensification may alter the structure of root microbial network and the abundance of keystone taxa, which in turn may have implications for crop performance [53, 54]. However, so far, it has not been investigated whether root microbial networks differ between organic, conservation, and conventional agriculture. A pertinent question is whether mycorrhizal fungi that are widely regarded for their role in plant productivity can also act as keystone taxa in the microbial community.

Here we explored the impact of farming systems on the fungal community structure using the latest *PacBio SMRT sequencing* and network analysis of wheat root samples collected from 60 farmlands in Switzerland. We aimed to address the following questions: (a) Does agricultural intensity affect the structure and composition of wheat root fungal communities? (b) Do network complexity and the abundance of keystone taxa vary between conventional, no-till, and organic farming? (c) Which taxa act as keystone and what are the drivers of such taxa in the root microbiota?

## Material and methods

### Site selection and sampling

Soil samples were collected in early May 2016 from wheat fields in 60 agricultural farmlands in the northeast and southwest regions of Switzerland (Figure S1). Wheat fields were either managed conventionally with tillage, conventionally under no-tillage, or organically under a mouldboard plough tillage for at least the last 5years. Farming systems were distributed equally in both regions, and each system was represented by 20 farmlands, resulting in a total of 60 farms. Conventionally managed fields applied pesticides and synthetic fertilizers and were managed following the ‘*Proof of Ecological Performance*’ guidelines of the Federal Office for Agriculture, Switzerland (https://www.blw.admin.ch). No-till fields were without any soil tillage except for occasional use of strip till, and potential application of synthetic substances (www.no-till.ch). Organically managed fields received no pesticides and synthetic fertilizers and were managed according to the guidelines of BioSuisse, the Federation of Swiss Organic Farmers (www.bio-suisse.ch). In addition to inherent differences among the farming systems in the use of plough or synthetic fertilizer and plant protection products, farmers also planted 25 different wheat varieties, all belonging to the list of recommended winter wheat varieties published annually by the Agrarforschung Schweiz (www.agrarforschungschweiz.ch) or BioSuisse, for conventionally or organically managed fields, respectively. While field sites showed a degree of variability in soil texture, elevation, and the mean annual temperature, none of these parameters differed significantly between the farming systems [55]. We calculated agricultural intensity index according to previous studies [54] based on the information collected from 59 farmers; information could not be obtained from one farm [55]. Agricultural intensity index was calculated using the information on three anthropogenic input factors: fertilizer use, pesticide use, and the consumption of fuel for agricultural machinery. These factors were also included in assessing agricultural intensity in a previous study.

At each farmland, 18 soil cores (4 cm diameter) were collected at 0–20 cm depth with a hand auger (Figure S2). These 18 samples were mixed and pooled to obtain a representative sample for a farm. The auger was cleaned between sites. Five undisturbed cylindrical soil cores of 100 ml volume and 5.1cm diameter were collected for bulk density measurement and the median of the five measures was considered as the estimate of bulk density for each field. Root samples were collected in June 2016 at wheat flowering (BBCH growth stage 69–75). At each site, ten wheat plants, five per transect, were excavated using a fork spade. Shoots were cut off at the height of ~5 cm and all roots of a specific site were pooled in a plastic bag for subsequent processing. Samples were placed on ice in a cooler box for transfer to the laboratory. Soil samples were processed on the same day as the collection by removing plant materials, homogenizing and passing through a 2-mm sieve. Sub-samples were taken for various soil physicochemical and biological analyses and stored at 4 °C or −20 °C as required.

### Plant and soil analyses

Root microbiome comprises microbial communities associated with plant roots, including microorganisms in the endosphere, rhizoplane, and rhizosphere [56–58]. This study specifically focused on the root endophytic fungal communities. In the lab, roots were thoroughly cleaned under cold tap water. Subsequently, fine roots (<1 mm) were cut into small pieces of about 1 cm length and thoroughly mixed. A subsample of 2 g of fine roots was stored in 1.5 Eppendorf tubes, lyophilized and stored at −20 °C for DNA extraction. The rest of the samples were used to determine AMF colonization by estimating the abundance of arbuscules, hyphae, or vesicles according to a modified line intersection method [59]. A minimum of 100 intersections per slide was examined with two technical replicates applying a blind procedure throughout the quantification process to avoid subjectivity related to the origin of the sample. For soil samples, total phosphorus (P), plant available P, pH, and bulk density were measured using the Swiss standard protocols [60]. Plant available P was measured according to Olsen et al. [61]. The abundance of AMF in soil was assessed by phospholipid fatty acid (PLFA) extraction followed by analysis on gas chromatography mass spectrometry [62]. We quantified the abundance of AMF in soil by using the PLFA 16:1ω5, which is well regarded as a biomarker for AMF because it constitutes a large proportion of total PLFAs in AMF, and strong correlations between AMF abundance in the soil and concentrations of the PLFA 16:1ω5 have been observed previously [63]. Neutral lipid fatty assay or NLFA 16:1ω5 is also used as an indicator of AMF biomass; however, NLFA 16:1ω5 is mainly present in storage organs [64]. Thus, it is considered a weak indicator of active AMF in soil and a previous study also found low amounts of NLFA 16:1ω5 in soil [65].

### DNA extraction and SMRT sequencing

For each sample, 200 mg of roots (dry weight) was used for DNA extraction using 600 mL of NucleoSpin lysis buffer PL1 for 15 min at 65 °C followed by the NucleoSpin Plant II kit (Macherey & Nagel, Düren, Germany). The DNA samples were amplified with the primer pair *ITS1F-ITS4* [66, 67] targeting the entire ITS region (~630 bp) [68]. The forward and reverse primers were synthesized with a 5-nucleotide-long padding sequence followed by barcode tags at the 5′ end to allow multiplexing of samples within a single sequencing run [69]. Library preparation and SMRT sequencing were conducted at the Functional Genomics Centre Zurich (http://www. fgcz.ch) on the PacBio® RS II Instrument (PacBio, San Diego, CA, USA). Details of PCR conditions and sequence data processing are described in the Supplementary Information. In brief, the SMRT Portal was used to extract the circular consensus sequences (CCS) from the raw data (available from the European Nucleotide Archive, study accession number: PRJEB27781). The CCS of at least five passes yield similar error rates as 454 or MiSeq sequencing platforms [68, 69]. The CCS reads were quality filtered in Mothur (v.1.35.0) [70]. Quality reads were demultiplexed based on the barcode-primer sequences using *flexbar* [71]. *De novo* chimera detection was performed on quality reads using UCHIME [72]. To avoid unwanted multi-primer artifacts, we deleted reads where full-length sequencing primer was detected within the read [73]. We clustered the quality sequences into operational taxonomic units (OTUs) at ≥98% sequence similarity with the UPARSE series of scripts [74]. Reads were de-replicated, and single-count and chimeric sequences were excluded for OTU delineation. The OTUs of low abundance (<0.1% global abundance and less than 0.5% abundance within a specific sample) were removed from the dataset (Figure S3). We normalized the OTU table by rarefying to 1000 reads per sample. On average 357 OTUs were found per site and a total of 837 OTUs for all 60 sites. The OTUs were classified taxonomically against the UNITE database [75]. The OTU and taxonomy tables were filtered to exclude OTUs classified as nonfungal.

### Statistical analyses

Alpha diversity indices such as OTU richness, Sheldon evenness and Shannon–Weaver index were calculated from the rarefied fungal OTU table using the *phyloseq* package [76] in R v3.4 [77]. The effect of farming systems and wheat varieties on fungal community structure was assessed by performing PERMANOVA and canonical analysis of principal coordinates with 999 permutations in PRIMER-E (PRIMER-E, Plymouth, UK). Fungal beta-diversity patterns were only assessed on OTUs that were present in at least two samples. Homogeneity of multivariate dispersions was checked with the PERMDISP test using the Bray–Curtis similarity matrix in PRIMER. We also identified the indicator taxa for each farming system using the ‘*multipatt*’ function in the *indicspecies* package in R [78]. Fundamentally, this analysis is based on two species traits: exclusivity (exclusively present in a habitat) and fidelity (present in all samples of that habitat) [79]. An indicator value is calculated based on these traits to assess the extent to which a species is indicator of a habitat.

Co-occurrence patterns in fungal communities were assessed by performing network analysis using the maximal information coefficient (MIC) scores in MINE statistics [80]. MIC is an insightful score that reveals positive, negative, and nonlinear associations among OTUs. Network analysis was performed on the same set of OTUs as testing for the beta-diversity i.e., only OTUs that were present in at least two samples were included, resulting in 822 OTUs. The overall meta-network was constructed with 60 samples, whereas the three farming-specific networks were constructed with 20 samples each. The MIC associations were corrected for false discovery rate (FDR) [81] and the final networks were constructed with relationships that were statistically significant (*P* < 0.05) after FDR correction. The networks were then visualized in Cytoscape version 3.4.0 [82]. The *NetworkAnalyzer* tool was used to calculate network topology parameters. Nodes (e.g., the fungal OTUs in this study) are the fundamental units of a network, while edges represent the connections or links between the nodes. Thus, degree represents the number of edges connected to a node. Clustering coefficient reflects the higher connectedness among nodes in a particular region of a network, whereas the shortest path indicates how quickly information can travel between two nodes [83]. Network diameter is the largest distance between two nodes of a network. We also evaluated networks against their randomized versions using the Barabasi–Albert model [84] available in *Randomnetworks* plugin in Cytoscape v2.6.1. Nodes in a random network may have the same number of degrees, resulting in a Poisson distribution. On the other hand, nonrandom networks are scale-free i.e., degree distribution shows a power-law tail with some nodes showing higher degrees than the rest [83]. Indeed, the structural attributes of root fungal networks such as degree distribution, mean shortest path, clustering coefficient were different from random networks with an equal number of nodes and edges. The OTUs with the highest degree and highest closeness centrality, and the lowest betweenness centrality scores were considered as the keystone taxa [48]. Closeness centrality is based on the average shortest paths and thus reflects the central importance of a node in disseminating information [85]. On the other hand, betweenness centrality reveals the role of a node as a bridge between components of a network. For the overall network, OTUs with degree greater than 50, closeness centrality higher than 0.44, and betweenness centrality lower than 0.12 were selected as the keystone taxa. For farming-specific networks, OTUs with degree higher than 10, closeness centrality higher than 0.28, and betweenness centrality lower than 0.18 were selected as the keystone taxa. We chose a single set of cut-off values for consistent comparison across farming-specific networks. We also calculated the proportional influence of various fungal orders in network structure by dividing the number of nodes belonging to a particular order by the number of connections (edges) it shared. This was based on the assumption that topological parameters have a direct influence on network structure [39]. We assessed the difference between farming-specific networks by bootstrapping node attributes (degree, between centrality, and closeness centrality) with 10,000 iterations. We then performed the two-sample Kolmogorov–Smirnov test to compare node attributes between farming systems using the *ks.test* function inbuilt in the *stats* package in R. Kolmogorov–Smirnov test compares the overall shape of the cumulative distribution of two variables where the null hypothesis is that the variables have same distribution patterns. For each network, node attributes were computed by bootstrapping approach with 10,000 iterations. Moreover, to compute node attributes for each farm, we used the *subgraph* function in the *igraph* package [86].

Finally, we performed random forest analysis to explore the determinants of the identified keystone taxa. Random Forest is a powerful machine learning tool that offers high prediction accuracy by using an ensemble of decision trees based on bootstrapped samples from a dataset [87]. It is a nonparametric and nonlinear statistical method that does not have prior distributional assumptions. The portion of dataset drawn into a sample is called *in-bag* data, whereas the data not drawn is termed as *out-of-bag* data [88]. Trees are fully grown to predict the *out-of-bag* data and the importance of a specific predictor variable is obtained by randomly permuting the values of that variable for the *out-of-bag* data and calculating increase in the mean squared error. Each node of a decision tree is associated with a subset of random data points from the original dataset and thus, increase in node purity (which is basically decrease in node impurity or misclassification rate) indicates the importance of a predictor variable. Random forest analysis was performed with 999 permutations using the *randomforest* and *rfPermute* packages [89]. The best predictors were identified based on their importance using the *importance* and *varImpPlot* functions. Increase in node purity and mean square error values were used to determine the significance of the predictors using the *randomForestExplainer* package [90]. The factors significant at *P* < 0.01 were selected as the predictors of keystone taxa.

## Results

### Overall structure and co-occurrence

**Fig. 1:**
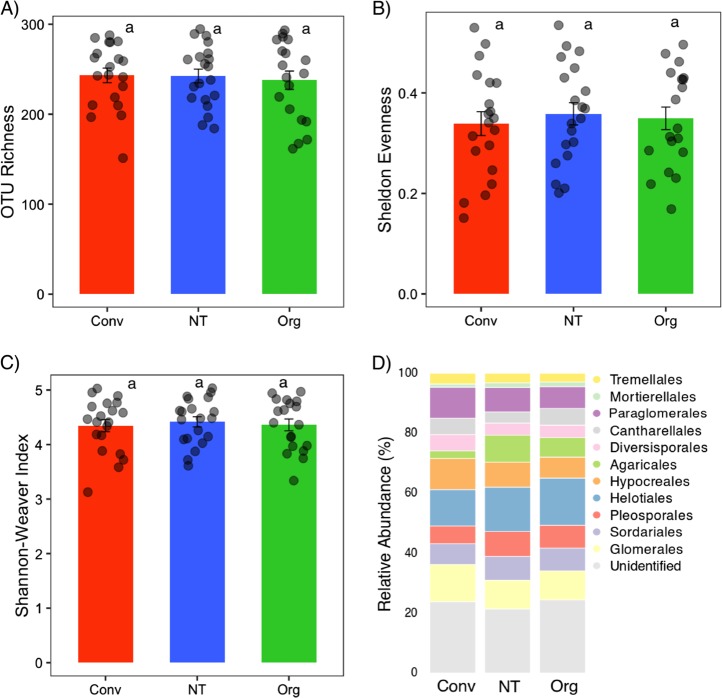
Alpha diversity indices and community composition of root fungal communities across conventional (Conv), no-till (NT), and organic (Org) farming systems. OTU richness (**a**), Sheldon evenness (**b**), and Shannon–Weaver index (**c**) were calculated from the rarefied fungal OTU table. Same lowercase letter indicates no statistically significant (*P* < 0.05) difference between farming systems. **d** Stacked bar chart showing the relative abundance of various orders of wheat root fungal communities

**Fig. 2:**
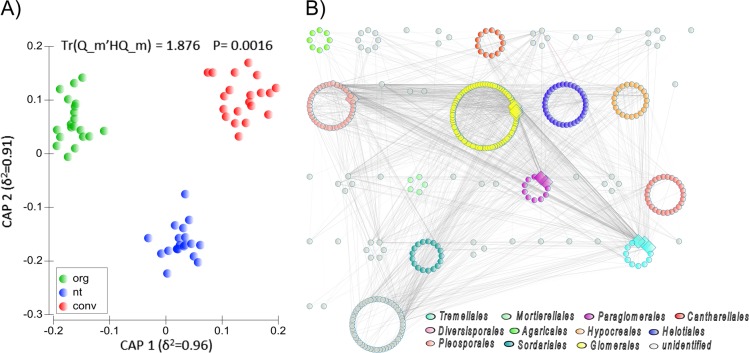
**a** Canonical analysis of principal coordinates (CAP) revealing a significant impact of farming systems on fungal community structure. **b** The overall network of root fungal communities across three farming systems. The overall network is arranged according to orders. White, red, and wavy lines represent positive, negative, and nonlinear relationships, respectively. Large diamond nodes indicate the keystone taxa in the network. Top ten nodes with the highest degree, highest closeness centrality, and lowest betweenness centrality were selected as the keystone taxa. Out of the ten keystone taxa in the overall network, seven belonged to mycorrhizal orders, *Glomerales, Paraglomerales*, and *Diversisporales*

Alpha diversity indices of root fungal communities did not vary significantly between the conventional, no-till, and organic systems (Fig. 1a-c). This was also true for the overall taxonomic composition (Fig. 1d). However, farming systems significantly influenced the root fungal community structure with three distinct clusters for organic, conventional, and no-tillage fields (Fig. 2a). A PERMANOVA test also confirmed the significant effect of farming systems (pseudo *F* = 1.42; *P* < 0.05; explained variation = 4.17%). A nonsignificant PERMDISP test (*F* = 2.072; *P* = 0.202) indicated homogenous dispersions of samples across systems. Further, a pairwise comparison in PERMDISP revealed that there was no significant difference in dispersions between organic and conventional (*F* = 1.068; *P* = 0.372), and organic and no-till (*F* = 0.870; *P* = 0.435). We found no impact of wheat varieties on community structure and this was reinforced by a nonsignificant PERMANOVA test (Pseudo *F* = 0.972; *P* = 0.595) (Figure S4). However, geographical locations i.e., northeast and southwest regions had an impact on root fungal community structure (Figure S5). Indicator species analysis was performed to test which taxa are characteristic for each of the three farming systems. Root inhabiting *Trichoderma*, a member of *Hypocreales*, was the only indicator taxon for conventional farming system, whereas seven fungal taxa (e.g., *Cyphellophora, Myrmecridium, Phaeosphaeria, Cadophora, Pyrenochaeta, Solicoccozyma*, and *Conocybe*) were the indicator taxa for no-till farming (Table S1). Six taxa of *Sordariales, Cantharellales*, and *Agaricales* were indicator taxa for organic farming with *Chaetomium* and *Psathyrella* as the only known genera.

The overall network of root fungal communities in 60 samples revealed distinct co-occurrence patterns (Fig. 2b). The meta-network consisted of 378 nodes and 1602 significant (*P* < 0.05) edges. This network with strong power-law distribution of degrees had a diameter of 8, average number of neighbors of 8.476, and a clustering coefficient of 0.258. For the overall network, eight of keystone taxa belonged to arbuscular mycorrhizal orders *Glomerales, Paraglomerales*, and *Diversisporales*, and the remaining five belonged to *Tremellales*, *Malasseziales*, and *Cantharellales* (Table S2). Indeed, the majority of the associations were from these four orders with *Glomerales* forming the largest guild with the maximum number of nodes and associations in the network. Overall, farming systems significantly affected fungal community structure with mycorrhizal orders playing a major role in the network complexity as measured by the number of edges, the average number of neighbors, and the clustering coefficient.

### Farming-specific co-occurrence networks

**Fig. 3:**
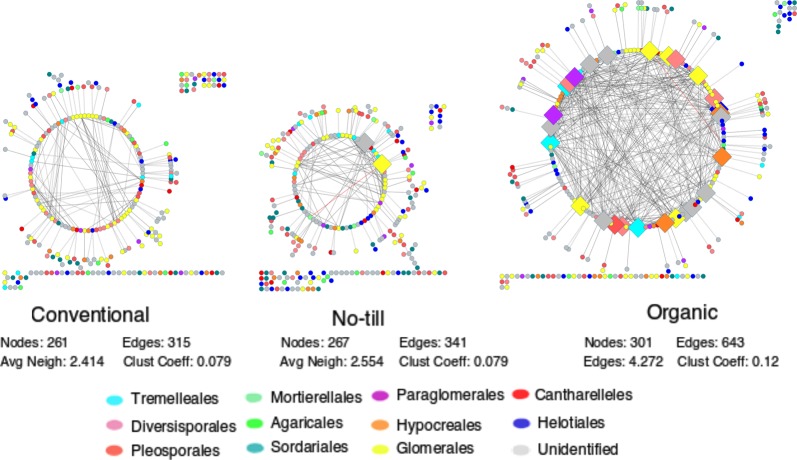
Farming system-specific root fungal networks. Each network was generated with root samples collected from 20 farmlands belonging to that farming system. The number of nodes, number of edges, average number of neighbors, and clustering coefficient is given below the specific networks. Large diamond nodes indicate the keystone taxa, whereas circular nodes indicate other taxa in the network. White, red, and wavy lines represent positive, negative, and nonlinear relationships, respectively. Despite having similar number of nodes, the organic network displayed twice more edges and many highly connected nodes than no-till and conventional networks that were dominated by less connected peripheral nodes

Owing to the significant difference in fungal community structure across three farming systems, we further evaluated root fungal networks for each farming system separately. The networks displayed remarkable differences in their structure and topology (Fig. 3). The network of conventional farming consisted of 261 nodes (e.g., taxa) and 315 edges (associations between taxa), while the no-till network consisted of 267 nodes and 341 edges. In stark contrast, the organic farming network consisted of 301 nodes and 643 edges. The average number of neighbors and the clustering coefficient of the organic farming network were also considerably higher than for the other two networks (Fig. 3). The higher complexity and connectivity in the organic farming network were supported by the abundance of keystone taxa. The organic farming network harbored 27 of such keystone taxa compared to two in the no-till network and none in the conventional one (Fig. 3; Table S3). The majority of these keystone taxa belonged to the orders *Glomerales*, *Tremellales,* and *Diversisporales* with a noticeable presence of taxa from the orders *Paraglomerales*, *Sebacinales,* and *Hypocreales*. To explore the importance of keystone taxa for the higher network complexity in organic farming, we constructed the organic network without including keystone OTUs. The organic network devoid of any keystone taxa was much simpler and was similar to the conventional and no-till networks (Figure S6).

**Fig. 4:**
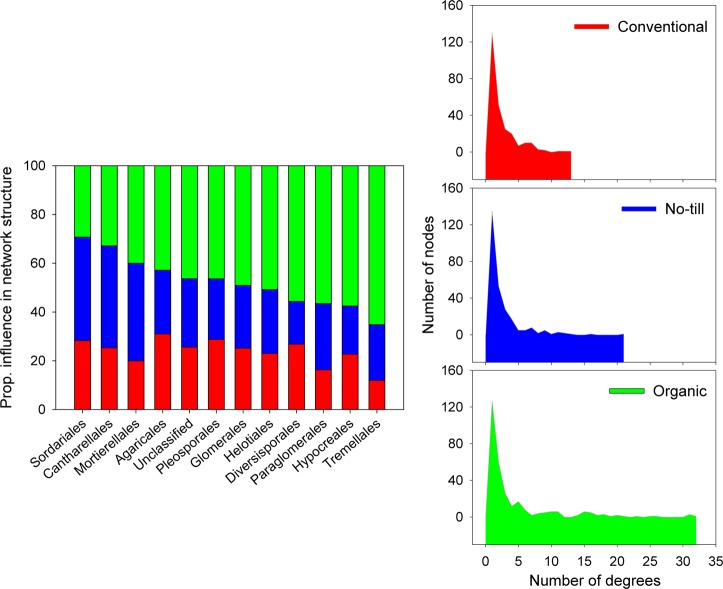
Proportional influence of various fungal orders in affecting the complexity of root microbiota (left panel). The influence was calculated by diving the number of nodes belonging to a particular fungal order by the number of connections (edges) it shared. It illustrates the orders that exhibit maximum connections across farming systems and thus influences network structure most. Distribution of degrees in three farming systems (right panel with three plots). Degree indicates the number of associations shared by each node in a network. In conventional, farming, the number of degrees was limited to a maximum of 12 compared to the no-till network that had a maximum of 22 degrees. On the other hand, organic farming had many nodes with over 20 degrees

Higher connectivity in the organic farming network was visible in the distribution of degrees, which indicates the number of associations shared by each node in a network (Fig. 4). The organic farming network had a much stronger power-law distribution than the conventional and no-till ones, despite the similar node distribution across root fungal orders (Figure S7). We calculated the proportional influence of various orders in the microbiota by dividing the number of nodes belonging to a particular order by the number of connections (edges) it shared. It revealed the orders that exhibited maximum connections across three farming systems and thereby influence the network structure. Various orders exhibited considerable differences in their proportional influence in the complexity of root microbiota. Orders such as *Sordariales* and *Agaricales* showed a major influence in the conventional network structure, and *Sordariales, Cantharellales*, and *Mortierellales* in the no-till network. In addition to *Tremellales and Hypocreales*, three mycorrhizal orders *Glomerales*, *Paraglomerales,* and *Diversisporales* showed a major influence on network complexity under organic farming. Overall, the organic farming network formed a much more complex network and harbored more keystone taxa than the other two farming networks.

### Drivers of keystone taxa

**Fig. 5:**
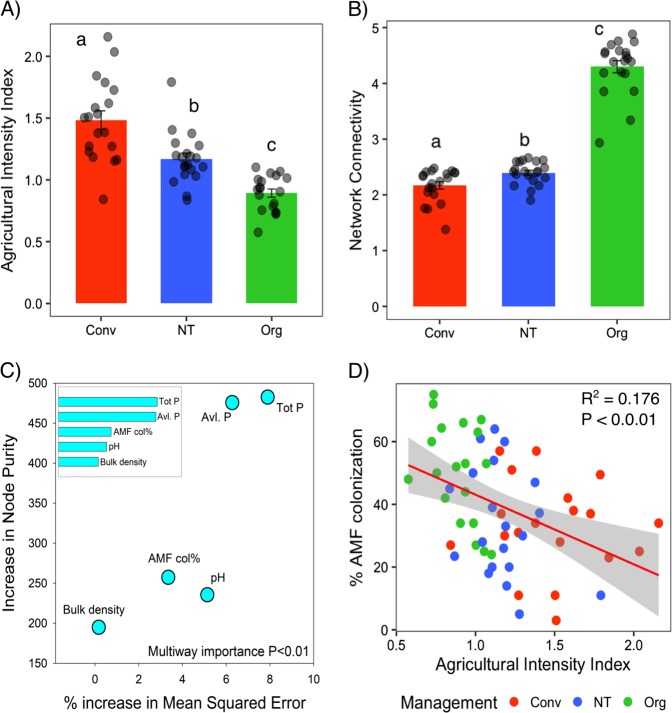
**a** Agricultural intensity index across conventional (Conv), no-till (NT), and organic (Org) farming systems. Agricultural intensity index was estimated using information on three anthropogenic input factors: fertilizer use, pesticide use, and the consumption of fuel for agricultural machineries. Different lowercase letters indicate statistically significant (*P* < 0.05) difference between farming systems. **b** Network connectivity as represented by node degrees for individual farms calculated by subsetting the networks of three farming systems. Different lowercase letters indicate statistically significant (P < 0.05) difference. **c** Results of random forest analysis showing the relative contribution of various factors in determining the abundance of keystone taxa. The mean squared error (MSE) indicates the prediction accuracy of each factor. The top (*P* < 0.01) five drivers were total phosphorus, plant available phosphorus (Olsen P), AMF root colonization, pH, and bulk density. **d** Relationship between agricultural intensification and mycorrhizal root colonization. Agricultural intensification had a significantly (*P* < 0.01) negative impact on the root colonization of AMF. Agricultural intensity was the highest under conventional farming and the lowest under organic farming, which was opposite for the AMF colonization

Agricultural intensity was significantly (*P* < 0.05) different across three farming practices with conventional being the most intensive and organic the least intensive system (Fig. 5a). This trend was opposite for network connectivity as represented by the node degree across three farming practices (Fig. 5b). Network bootstrapping revealed that the network connectivity in organic fields was significantly (*P* < 0.05) higher than the conventional and no-till ones. Kolmogorov–Smirnov test showed that node degree, betweenness centrality, and closeness centrality were significantly (*P* < 0.01) different between the three framing systems (Table S4). Moreover, network connectivity was inversely proportional to agricultural intensity index (*R*^2^ = 0.366; *P* < 0.0001; Figure S9). The number of keystone taxa was also higher (27) in the organic farming network than the no-till (2) conventional (0) networks. Random forest analysis revealed that soil phosphorus content, bulk density, pH, and mycorrhizal colonization best explained (*P* < 0.01) the occurrence of keystone taxa (Fig. 5c). Most of these parameters were also significantly (*P* < 0.05) correlated with the alpha-diversity indices, indicating their importance for the overall root fungal communities (Table S5). The majority of keystone taxa belonged to mycorrhizal orders, and mycorrhizal colonization of wheat roots was significantly (*P* < 0.01) higher in the organic fields than in the conventional and no-till fields (Figure S8). Consistent with this, the abundance of mycorrhizal PLFA in soil was also significantly (*P* < 0.01) higher in the organic compared to the conventional fields. Agricultural intensity had a significantly negative impact on mycorrhizal colonization in roots and the abundance in soils (Fig. 5d). Taken together, the root fungal network complexity, abundance of keystone taxa and mycorrhizal abundance showed an opposite trend to that of agricultural intensification across farming systems.

## Discussion

It is now well established that root-associated microbiota plays an important role in plant diversity, community composition, and performance [24, 35, 58, 91]. Consequently, it is important to understand how microbial communities harbored inside crop roots are affected by agricultural practices and how key microbial players can be targeted for ecological intensification of agroecosystems [5]. However, with much of the previous work only focusing on the soil microbiota, our understanding of the effects of farming systems on root-associated microbiota is still rudimentary. Moreover, previous studies mostly focused on microbial alpha- and beta-diversity patterns, and the impact of different farming systems on microbial network structure is poorly understood. Here we show that wheat roots under different farming systems harbor distinct fungal communities and with varying network complexity. Fungal network complexity of organically managed fields was almost twice as high under conventional and no-till farming practices. Moreover, network connectivity was negatively associated with agricultural intensification.

Our finding that the overall structure of root microbiota influenced by farming systems is in agreement with studies on the soil microbiome where a large number of reports showed a significant impact of farming systems [6, 20–22, 92, 93]. It should be noted that most of these studies investigated microbial communities in agronomical context and were performed in field-trials [20–22, 32]. While a major strength of field-trials is that farming treatments are imposed under homogenous management and at one location with a specific soil type, management effects on microbial patterns may be different in actual farmlands and thus the results obtained at one location may not be generalized. We report the impact of farming practices on root microbial community characteristics in on-farm research and across many fields at a regional scale.

Microorganisms do not thrive in isolation and rather form complex association networks. Such networks hold special importance for gaining insight into microbiome structure and its response to environmental factors [25, 42, 43, 47, 51]. Our study highlights how farming practices impact the network structure of root microbiota and uncovers that organic farming harbors a significantly more complex network with many highly connected taxa (nodes) than the conventional and no-till farming. It has been shown that complex networks with greater connectivity are more robust to environmental perturbations than simple networks with lower connectivity [94]. In this sense, the higher complexity of organic networks may indicate that the root microbiota under organic management is more resilient to environmental stresses as different taxa can complement each other. However, further studies are necessary to corroborate this observation.

Keystone taxa are the highly connected taxa that play important roles in the microbiome and their removal can cause significant changes in microbiome composition and functioning [48, 50]. Although previous studies have reported keystone taxa in various environments [34, 45, 95], reports on keystone taxa in the root endophytic microbiota are very limited. The organic farming network exhibited by far the highest connectivity and comprised most of the keystone taxa. It should be noted that fungal richness did not vary significantly between the farming systems nor did the number of nodes across farming-specific networks, and yet we observed a clear difference in the network structure and number of keystone OTUs. Moreover, the abundance of keystone OTUs did not vary between the three farming systems but these OTUs shared considerably more associations in organic farming (Figure S10). The organic network without the keystone OTUs was similar to the conventional and no-till networks, highlighting the importance of these members for network complexity. Our observations indicate that microbiome complexity is not necessarily determined by the number of taxa in the community, but rather the number of associations that those taxa share amongst them.

The majority of these keystone taxa were AMF belonging to the orders *Diversisporales, Glomerales*, and *Paraglomerales*. The symbiotic association of AMF that started more than 400 million years ago is formed by ~80% of terrestrial plants [36, 96]. The observation that AMF can enhance plant productivity [97] make them a crucial player in agroecosystems. The importance of AMF for the root-associated microbiota, particularly under organic farming, is congruent with the higher abundance of AMF in roots and soils observed in the organic farmlands in this study (Figure S8). While previous studies also found significantly higher AMF abundance and diversity in organic farmlands than in the conventional ones [98, 99], the important role of AMF for the root fungal network structure is reported here. One of the nonmycorrhizal keystone taxa in organic farming belonged to the order *Sebacinales*. Members of this order are highly diverse root endophytes and are thought to form neutral and beneficial interactions with plants [100]. Our observation of *Sebacinales* as keystone taxa is consistent with a previous report that found a consistently higher abundance of *Sebacinales* in organic farmlands [31]. Since keystone taxa are linked to network complexity, beneficial endophytic keystone taxa such as AMF and *Sebacinales* may enhance the network connectivity and thereby the complexity of the root microbiome. Several other keystone taxa in the overall and organic networks belonged to the order *Tremellales*. This widespread group of Basidiomycetes contains many yeast species and have been reported in plant roots in temperate regions [101]. Members of this fungal order were also recently found as keystone taxa in the root microbiome across eight forest ecosystems in Japanese Archipelago [53]. Interestingly, we found that two of the keystone taxa (OTU_10, OTU_11) were members of the *Dioszegia* genus, which was also found as keystone by Agler et al. [51]. It was shown that the effect of abiotic factors on microbiome was mediated via *Dioszegia* in *Arabidopsis thaliana*. The consistent identification of *Dioszegia* as a keystone taxon across studies suggests its importance and highlights a potential that it can be harnessed for manipulation of the plant microbiome. Future studies are now needed to specifically manipulate this group to test how it influences microbiome composition and functioning. There were no common fungal groups between indicator taxa and keystone taxa. It should be noted that indicator taxa are identified based on their exclusive abundance (exclusivity) in all samples (fidelity) under a particular habitat [79], whereas keystone taxa are identified using a network algorithm that focuses on the number of associations an OTU shares and its position in the microbiome [48]. Thus, indicator taxa and keystone taxa reflect two different microbial indices that target different members in the community.

An important question is how do farming practices and land use intensity affect the structure and network complexity of the root endophytic fungi? We speculate that there might be two underlying mechanisms: the assembly of fungal members in the soil, and their recruitment and colonization of the plant root. It is well known that farming practices affect the quality and quantity of important soil nutrients such as carbon, nitrogen, and phosphorus [6, 8, 102, 103]. Reduced or no-tillage can also alter the bulk density in the topsoil with subsequent impact on the root architecture and elongation [28]. These factors can modulate the assembly and evolution of microbes in the soil [29, 104–106], thereby affecting microbial recruitment into the root. Indeed, we found soil phosphorus levels, bulk density and also pH to be the determinants of keystone taxa, which are linked to network complexity. The majority of keystone taxa were mycorrhizal in nature, and phosphorus is well acknowledged for its importance for mycorrhizal associations [107]. Similarly, soil pH is a known driver of fungal communities in soil, especially, mycorrhizal fungi [108, 109]. Thus, the identification of soil characteristic as the determinants of keystone taxa indicates the importance of recruitment as a driver of network complexity of the root endophytic microbiota.

Once recruited inside the plant body, microbial adaptation and survival will depend on the host physiological patterns [26, 58, 110]. Farming practices may also influence crop physiological responses via water and nutrient availability, and pesticide application [103, 111, 112], which can affect the maintenance of endophytic microbes inside the plant body. For example, it is known that crops are able to reduce carbon allocation to mycorrhizal fungi when grown under high nutrient availability due to agricultural intensification [29]. Host genotypes may also affect plant physiological responses and endophytic microbiota, although in this study, we did not find a clear link between wheat varieties and root fungi. However, our field sites had different wheat varieties growing, and whether or not host genotypes influence root fungal community structure would require a site-specific experiment with multiple varieties growing under one field condition, which was beyond the scope of this study. Previous studies also found that soil conditions had a stronger effect on root fungal communities than host species, while the opposite was true for bacterial communities [113, 114]. Such mixed results highlight the complex nature of plant–microbe interactions [115] and the need for further research targeting the factors influencing crop endophytic microbial communities under different farming practices. Moreover, soil and plant sampling in this study were only conducted for one year, and thus repeated sampling would be the next step to assess the temporal consistency and predictability of these findings.

While the exact drivers of network complexity of root endophytes remain unknown, it is possible that nutritional status, tillage, manure application, and the absence of pesticides might have created unique environments in each of the three farming practices, potentially influencing the assembly of fungi in the soil and their recruitment into the plant root. Large amounts of chemical fertilizers in the conventional farming system may foster fast-growing (*r-strategists*) microbes without strong selection pressure for any particular taxa and thus, creating a more random assemblage. In contrast, the application of organic amendments with lower immediate resource availability may act as a selective force on the assembly of fungal communities, promoting slow-growing (*K-strategists*) microbes [116]. It is possible that microbial communities under organic farming may be dominated by the *K-strategists* that establish themselves slower and have a higher chance to coevolve. For such microbial communities occurring under resource-limited conditions, microbial cooperation may be more important for survival. Cooperation requires a high degree of connectivity, leading to networks with higher complexity. Microbial communities with higher network complexity may thus be more common under extensive management where inputs are low and resources are limited, which accords with a recent study on grasslands [47]. The number of keystone taxa was indeed the highest under organic farming where agricultural intensity was the lowest, and we also found a significantly strong negative association between agricultural intensification and network connectivity. Nonetheless, it should be noted that microbial taxa associating in a co-occurrence network may not be due to their actual interaction [41, 117]. Furthermore, we only considered root fungi in this study, and a microbiome comprises bacteria, archaea, and other members, the inclusion of which is necessary for gaining insight into root microbial network structure. It is also important to mention that identification of keystone taxa are based on the analysis of correlations (associations) among taxa, and further research is necessary to show the causality, in terms of the impact of keystone taxa on microbiome structure and functioning.

## Conclusions

The structure and composition of root microbiota play an important role in agroecosystems and yet there is a significant dearth of knowledge about the effect of agricultural intensification on the root microbiota. van der Heijden and Hartmann [106] highlighted the importance of network structure for the functioning of plant microbiomes while Banerjee et al. [49] recently summarized keystone taxa from various environments to emphasize their importance for microbiome structure and functioning. The present study builds on and extends this conceptual framework to demonstrate that the agricultural intensification has a negative influence on root fungal network structure and the abundance of keystone taxa. Our study shows that the network connectivity and the abundance of keystone taxa were the highest under organic farming where agricultural intensity was the lowest. The higher co-occurrence of members of microbial communities under organic farming may be indicative of greater ecological balance and complexity of the microbiome, which might be more resilient to environmental stresses. A key strength of this study is that the samples were collected from 60 farmlands and the reported effects can be generalized because samples were taken from an extensive range of fields at different locations with different management regimes. The recent concept of smart farming (Wolfert et al. [118]) emphasizes *thinking outside the box*. The potential for harnessing plant microbiome for sustainable agriculture was also highlighted recently [119]. Mycorrhizal fungi are well regarded for their effects on plant productivity, and thus mycorrhizal keystone taxa may be targeted as a tool for smart farming.

## Supplementary information

Supplementary Information

R script

## References

[1] Foley JA, Ramankutty N, Brauman KA, Cassidy ES, Gerber JS, Johnston M (2011). Solutions for a cultivated planet. Nature.

[2] FAO. Food and Agriculture Organization of the United Nations. 2018. http://www.fao.org. Accessed 22 Mar 2018.

[3] Tilman D, Cassman KG, Matson PA, Naylor R, Polasky S (2002). Agricultural sustainability and intensive production practices. Nature.

[4] Foley JA (2005). Global consequences of land use. Science.

[5] Bender SF, Wagg C, van der Heijden MGA (2016). An underground revolution: biodiversity and soil ecological engineering for agricultural sustainability. Trends Ecol Evol.

[6] Lori M, Symnaczik S, Mäder P, De Deyn G, Gattinger A (2017). Organic farming enhances soil microbial abundance and activity—A meta-analysis and meta-Regression. PLoS ONE.

[7] Hobbs PR, Sayre K, Gupta R (2008). The role of conservation agriculture in sustainable agriculture. Philos Trans R Soc B Biol Sci.

[8] Martínez-García LB, Korthals G, Brussaard L, Jørgensen HB, De Deyn GB (2018). Organic management and cover crop species steer soil microbial community structure and functionality along with soil organic matter properties. Agric Ecosyst Environ.

[9] Willer H, Schaack D, Lernoud J (2017). Organic farming and market development in Europe and the European Union. The world of organic agriculture-statistics and emerging trends 2017.

[10] Soane BD, Ball BC, Arvidsson J, Basch G, Moreno F, Roger-Estrade J (2012). No-till in northern, western and south-western Europe: A review of problems and opportunities for crop production and the environment. Soil Tillage Res.

[11] van der Heijden MGA, Bardgett RD, Van Straalen NM (2008). The unseen majority: Soil microbes as drivers of plant diversity and productivity in terrestrial ecosystems. Ecol Lett.

[12] Bardgett RD, van der Putten WH (2014). Belowground biodiversity and ecosystem functioning. Nature.

[13] Delgado-Baquerizo M, Maestre FT, Reich PB, Jeffries TC, Gaitan JJ, Encinar D (2016). Microbial diversity drives multifunctionality in terrestrial ecosystems. Nat Commun.

[14] Fierer N (2017). Embracing the unknown: disentangling the complexities of the soil microbiome. Nat Rev Microbiol.

[15] Graham EB, Knelman JE, Schindlbacher A, Siciliano S, Breulmann M, Yannarell A (2016). Microbes as engines of ecosystem function: When does community structure enhance predictions of ecosystem processes?. Front Microbiol.

[16] Wall DH, Nielsen UN, Six J (2015). Soil biodiversity and human health. Nature.

[17] Philippot L, Raaijmakers JM, Lemanceau P, Van Der Putten WH (2013). Going back to the roots: The microbial ecology of the rhizosphere. Nat Rev Microbiol.

[18] Matson PAa, Parton WJJ, Power AGG, Swift MJJ (1997). Agricultural intensification and ecosystem properties. Science.

[19] de Vries FT, Thebault E, Liiri M, Birkhofer K, Tsiafouli MA, Bjornlund L (2013). Soil food web properties explain ecosystem services across European land use systems. Proc Natl Acad Sci USA.

[20] Hartmann M, Frey B, Mayer J, Mäder P, Widmer F (2015). Distinct soil microbial diversity under long-term organic and conventional farming. ISME J.

[21] Lupatini M, Korthals GW, de Hollander M, Janssens TKS, Kuramae EE (2017). Soil microbiome is more heterogeneous in organic than in conventional farming system. Front Microbiol.

[22] Schmidt R, Gravuer K, Bossange AV, Mitchell J, Scow K (2018). Long-term use of cover crops and no-till shift soil microbial community life strategies in agricultural soil. PLoS ONE.

[23] de Vries FT, Wallenstein MD (2017). Below-ground connections underlying above-ground food production: a framework for optimising ecological connections in the rhizosphere. J Ecol.

[24] Fitzpatrick Connor R., Copeland Julia, Wang Pauline W., Guttman David S., Kotanen Peter M., Johnson Marc T. J. (2018). Assembly and ecological function of the root microbiome across angiosperm plant species. Proceedings of the National Academy of Sciences.

[25] Edwards J, Johnson C, Santos-Medellín C, Lurie E, Podishetty NK, Bhatnagar S (2015). Structure, variation, and assembly of the root-associated microbiomes of rice. Proc Natl Acad Sci USA.

[26] Hardoim PR, van Overbeek LS, Elsas JDvan (2008). Properties of bacterial endophytes and their proposed role in plant growth. Trends Microbiol.

[27] Unger PW, Kaspar TC (1994). Soil compaction and root growth: a review. Agron J.

[28] Seghers D, Wittebolle L, Top EM, Verstraete W, Siciliano SD (2004). Impact of agricultural practices on the Zea mays L. endophytic community. Appl Environ Microbiol.

[29] Oehl F, Sieverding E, Ineichen K, Mäder P, Boller T, Wiemken A (2003). Impact of land use intensity on the species diversity of arbuscular mycorrhizal fungi in agroecosystems of central europe impact of land use intensity on the species diversity of arbuscular mycorrhizal fungi in agroecosystems of Central Europe. Appl Environ Microbiol.

[30] Opik M, Moora M, Liira J, Zobel M (2006). Composition of root-colonizing arbuscular mycorrhizal fungal communities in different ecosystems around the globe. J Ecol.

[31] Verbruggen E, Rillig MC, Wehner J, Hegglin D, Wittwer R, van der Heijden MGA (2014). Sebacinales, but not total root associated fungal communities, are affected by land-use intensity. New Phytol.

[32] Hartman K, van der Heijden MGA, Wittwer RA, Banerjee S, Walser JC, Schlaeppi K (2018). Cropping practices manipulate abundance patterns of root and soil microbiome members paving the way to smart farming. Microbiome.

[33] Vandenkoornhuyse P, Baldauf SL, Leyval C, Straczek J, Young JPW (2002). Extensive fungal diversity in plant roots. Science.

[34] Shi S, Nuccio EE, Shi ZJ, He Z, Zhou J, Firestone MK (2016). The interconnected rhizosphere: High network complexity dominates rhizosphere assemblages. Ecol Lett.

[35] Bennett JA, Maherali H, Reinhart KO, Lekberg Y, Hart MM, Klironomos J (2017). Plant-soil feedbacks andmycorrhizal type influence temperate forest population dynamics. Science.

[36] Smith JE, Read DJ (2008). Mycorrhizal symbiosis.

[37] Gill SS, Gill R, Trivedi DK, Anjum NA, Sharma KK, Ansari MW (2016). Piriformospora indica: Potential significance plant stress toler.

[38] Harman GE, Howell CR, Viterbo A, Chet I, Lorito M (2004). Trichoderma species - Opportunistic, avirulent plant symbionts. Nat Rev Microbiol.

[39] Strogatz SH (2001). Exploring complex networks. Nature.

[40] Fuhrman JA (2009). Microbial community structure and its functional implications. Nature.

[41] Faust K, Raes J (2012). Microbial interactions: from networks to models. Nat Rev Microbiol.

[42] de Vries FT, Griffiths RI, Bailey M, Craig H, Girlanda M, Gweon HS (2018). Soil bacterial networks are less stable under drought than fungal networks. Nat Commun.

[43] Ramirez KS, Geisen S, Morriën E, Snoek BL, van der Putten WH (2018). Network analyses can advance above-belowground ecology. Trends Plant Sci.

[44] Barberán A, Bates ST, Casamayor EO, Fierer N (2012). Using network analysis to explore co-occurrence patterns in soil microbial communities. ISME J.

[45] Ma B, Wang H, Dsouza M, Lou J, He Y, Dai Z (2016). Geographic patterns of co-occurrence network topological features for soil microbiota at continental scale in eastern China. ISME J.

[46] Leff JW, Bardgett RD, Wilkinson A, Jackson BG, Pritchard WJ, de Long JR, et al. Predicting the structure of soil communities from plant community taxonomy, phylogeny, and traits. ISME J. 2018;1–12. 10.1038/s41396-018-0089-x PMC6004312 29523892

[47] Morriën E, Hannula E, Snoek LB, Helmsing NR, Zweers H, De Hollander M (2017). Soil networks become more connected and take up more carbon as nature restoration progresses. Nat Commun.

[48] Berry D, Widder S (2014). Deciphering microbial interactions and detecting keystone species with co-occurrence networks. Front Microbiol.

[49] Banerjee S, Schlaeppi K, van der Heijden MGA (2018). Keystone taxa as drivers of microbiome structure and functioning. Nat Rev Microbiol.

[50] Herren CM, McMahon KD (2018). Keystone taxa predict compositional change in microbial communities. Environ Microbiol.

[51] Agler MT, Ruhe J, Kroll S, Morhenn C, Kim ST, Weigel D (2016). Microbial hub taxa link host and abiotic factors to plant microbiome variation. PLoS Biol.

[52] Durán P, Thiergart T, Garrido-Oter R, Agler M, Kemen E3, Schulze-Lefert P (2018). Microbial interkingdom interactions in roots promote Arabidopsis survival. Cell.

[53] Toju H, Tanabe AS, Sato H (2018). Network hubs in root-associated fungal metacommunities. Microbiome.

[54] Wittwer RA, Dorn B, Jossi W, Van Der Heijden MGA (2017). Cover crops support ecological intensification of arable cropping systems. Sci Rep.

[55] Büchi L, Georges F, Walder F, Banerjee S, Keller T, Six J, et al. Potential of indicators to unveil the hidden side of cropping system classification: actual differences and similarities in cropping practices between conventional, no-till and organic systems. (Submitted) 2019.

[56] Sasse J, Martinoia E, Northen T (2017). Feed your friends: do plant exudates shape the root microbiome?. Trends Plant Sci.

[57] Bulgarelli D, Schlaeppi K, Spaepen S, van Themaat EVL, Schulze-Lefert P (2013). Structure and functions of the bacterial microbiota of plants. Annu Rev Plant Biol.

[58] Hardoim PR, van Overbeek LS, Berg G, Pirttilä AM, Compant S, Campisano A (2015). The hidden world within plants: ecological and evolutionary considerations for defining functioning of microbial endophytes. Microbiol Mol Biol Rev.

[59] McGonigle TP, Miller MH, Evans DG, Fairchild GL, Swan JA (1990). A new method which gives an objective measure of colonization of roots by vesicular- arbuscular mycorrhizal fungi. New Phytol.

[60] FAL, FAW, RAC. Referenzmethoden der Eidg. landwirtschaftlichen Forschungsanstalten. 1. Bodenuntersuchung zur Du¨ngeberatung, Zu¨rich-Reckenholz. 1996.

[61] Olsen SR, Cole CV, Watanabe FS, Dean L (1954). Estimation of available phosphorus in soils by extraction with sodium bicarbonate.

[62] Esperschütz J, Buegger F, Winkler JB, Munch JC, Schloter M, Gattinger A (2009). Microbial response to exudates in the rhizosphere of young beech trees (Fagus sylvatica L.) after dormancy. Soil Biol Biochem.

[63] Olsson PA, Francis R, Read DJ (1998). Growth of arbuscular mycorrhizal mycelium in calcareous dune sand and its interaction with other soil microorganisms as estimated by measurement of specific fatty acids. Plant Soil.

[64] Verbruggen E, Jansa J, Hammer EC, Rillig MC (2016). Do arbuscular mycorrhizal fungi stabilize litter-derived carbon in soil?. J Ecol.

[65] Olsson P, Thingstrup I, Jakobsen I, Bååth E (1999). Estimation of the biomass of arbuscular mycorrhizal fungi in a linseed field. Soil Biol Biochem.

[66] White TJ, Bruns TD, Lee SB, Taylor JW, Innis MA, Gelfand DH, Sninsky JJWT (1990). PCR protocols: a guide to methods and applications. PCR protocols: a guide to methods and applications..

[67] Gardes M, Bruns TD (1993). ITS primers with enhanced specificity for basidiomycetes, application to the identification of mycorrihiza and rusts. Mol Ecol.

[68] Bodenhausen N, Somerville V, Desiro A, Walser J-C, Borghi L, Heijden M van der, et al. Species-specific root microbiota dynamics in response to plant-available phosphorus. bioRxiv. 2018. https://doi.org/10.1101/400119.

[69] Schlaeppi K, Bender SF, Mascher F, Russo G, Patrignani A, Camenzind T (2016). High-resolution community profiling of arbuscular mycorrhizal fungi. New Phytol.

[70] Schloss PD, Westcott SL, Ryabin T, Hall JR, Hartmann M, Hollister EB (2009). Introducing mothur: Open-source, platform-independent, community-supported software for describing and comparing microbial communities. Appl Environ Microbiol.

[71] Dodt M, Roehr J, Ahmed R (2012). Dieterich C. FLEXBAR—Flexible barcode and adapter processing for next-generation sequencing platforms. Biol (Basel).

[72] Edgar RC, Haas BJ, Clemente JC, Quince C, Knight R (2011). UCHIME improves sensitivity and speed of chimera detection. Bioinformatics.

[73] Tedersoo L, Tooming-Klunderud A, Anslan S (2018). PacBio metabarcoding of Fungi and other eukaryotes: errors, biases and perspectives. New Phytol.

[74] Edgar RC (2013). UPARSE: highly accurate OTU sequences from microbial amplicon reads. Nat Methods.

[75] Koljalg U, Nilsson RH, Abarenkov K, Tedersoo L, Taylor AFS, Bahram M (2013). Towards a unified paradigm for sequence-based identification of fungi. Mol Ecol.

[76] McMurdie PJ, Holmes S (2013). Phyloseq: An R package for reproducible interactive analysis and graphics of microbiome census data. PLoS ONE.

[77] R Core Team. R: A language and environment for statistical computing. Vienna, Austria: R Foundation for Statistical Computing; 2017.

[78] De Cáceres M, Jansen F. Package ‘indicspecies’ (Version 1.7.6). 2016. http://cran.r-project.org/web/packages/indicspecies/indicspecies.pdf

[79] Dufrene M, Legendre P (1997). Species assemblages and indicator species: the need for a flexible asymmetrical approach. Ecol Monogr.

[80] Reshef DN, Reshef YA, Finucane HK, Grossman SR, McVean G, Turnbaugh PJ (2011). Detecting novel associations in large data sets. Science.

[81] Benjamini Y, Hochberg Y (1995). Controlling the false discovery rate: a practical and powerful approach to multiple testing. J R Stat Soc B.

[82] Shannon P, Markiel A, Ozier O, Baliga NS, Wang JT, Ramage D (2003). Cytoscape: A software environment for integrated models of biomolecular interaction networks. Genome Res.

[83] Barabási AL, Gulbahce N, Loscalzo J (2011). Network medicine: a network-based approach to human disease. Nat Rev Genet.

[84] Barabasi AL, Albert R (1999). Emergence of scaling in random networks. Science.

[85] Freeman LC (1978). Centrality in social networks conceptual clarification. Soc Netw.

[86] Csárdi G, Nepusz T (2006). The igraph software package for complex network research. InterJournal Complex Syst.

[87] Breiman L (2001). Random forests. Mach Learn.

[88] Prasad AM, Iverson LR, Liaw A (2006). Newer classification and regression tree techniques: Bagging and random forests for ecological prediction. Ecosystems.

[89] Archer E. Package ‘ rfPermute’. https://cran.r-project.org/web/packages/rfPermute/index.html. 2013. Accessed 5 Oct 2017.

[90] Paluszynska A, Biecek P. Package ‘randomForestExplainer’. Explaining and visualizing random forests in terms of variable importance. 2017. https://cran.r-project.org/web/packages/randomForestExplainer/index.html.

[91] Teste FP, Kardol P, Turner BL, Wardle DA, Zemunik G, Renton M (2017). Plant-soil feedback and the maintenance of diversity in Mediterranean-climate shrublands. Science.

[92] Postma-Blaauw MB, De Goede RGM, Bloem J, Faber JH, Brussaard L (2010). Soil biota community structure and abundance under agricultural intensification and extensification. Ecology.

[93] Wagg C, Dudenhöffer JH, Widmer F, van der Heijden MGA (2018). Linking diversity, synchrony and stability in soil microbial communities. Funct Ecol.

[94] Santolini M, Barabási A (2018). Predicting perturbation patterns from the topology of biological networks. Proc Natl Acad Sci USA.

[95] Banerjee S, Kirkby CA, Schmutter D, Bissett A, Kirkegaard JA, Richardson AE (2016). Network analysis reveals functional redundancy and keystone taxa amongst bacterial and fungal communities during organic matter decomposition in an arable soil. Soil Biol Biochem.

[96] Strullu-Derrien C, Selosse MA, Kenrick P, Martin FM (2018). The origin and evolution of mycorrhizal symbioses: From palaeomycology to phylogenomics. New Phytol.

[97] van der Heijden MG, Bruin SDe, Luckerhoff L, van Logtestijn RS, Schlaeppi K (2016). A widespread plant-fungal-bacterial symbiosis promotes plant biodiversity, plant nutrition and seedling recruitment. ISME J.

[98] Verbruggen E, Röling WFM, Gamper Ha, Kowalchuk Ga, Verhoef Ha, Heijden MGaVanDer (2010). Positive effects of organic farming on below-ground mutualists: large-scale comparison of mycorrhizal fungal comparison in agricultural soils. New Phytol.

[99] Oehl F, Sieverding E, Mäder P, Dubois D, Ineichen K, Boller T (2004). Impact of long-term conventional and organic farming on the diversity of arbuscular mycorrhizal fungi. Oecologia.

[100] Weiss M, Waller F, Zuccaro A, Selosse MA (2016). Sebacinales - one thousand and one interactions with land plants. New Phytol.

[101] Gao Q, Yang ZL (2016). Diversity and distribution patterns of root-associated fungi on herbaceous plants in alpine meadows of southwestern China. Mycologia.

[102] McDaniel MD, Tiemann LK, Grandy AS (2014). Does agricultural crop diversity enhance soil microbial biomass and organic matter dynamics? A meta-analysis. Ecol Appl.

[103] Haling RE, Simpson RJ, Delhaize E, Hocking PJ, Richardson AE (2010). Effect of lime on root growth, morphology and the rhizosheath of cereal seedlings growing in an acid soil. Plant Soil.

[104] Donn S, Kirkegaard JA, Perera G, Richardson AE, Watt M (2015). Evolution of bacterial communities in the wheat crop rhizosphere. Environ Microbiol.

[105] Germida JJ, Siciliano SD, De Freitas JR, Seib AM (1998). Diversity of root-associated bacteria associated with field-grown canola (Brassica napus L.) and wheat (Triticum aestivum L.). FEMS Microbiol Ecol.

[106] van der Heijden MGA, Hartmann M (2016). Networking in the plant microbiome. PLoS Biol.

[107] Powell Jeff R., Rillig Matthias C. (2018). Biodiversity of arbuscular mycorrhizal fungi and ecosystem function. New Phytologist.

[108] Rousk J, Bååth E, Brookes PC, Lauber CL, Lozupone C, Caporaso JG (2010). Soil bacterial and fungal communities across a pH gradient in an arable soil. ISME J.

[109] Nilsson LO, Bååth E, Falkengren-Grerup U, Wallander H (2007). Growth of ectomycorrhizal mycelia and composition of soil microbial communities in oak forest soils along a nitrogen deposition gradient. Oecologia.

[110] Edwards JA, Santos-Medellín CM, Liechty ZS, Nguyen B, Lurie E, Eason S (2018). Compositional shifts in root-associated bacterial and archaeal microbiota track the plant life cycle in field-grown rice. PLoS Biol.

[111] Jones VP, Toscano NC, Johnson MW, Welter SC, Youngman RR (1986). Pesticide effects on plant physiology: integration into a pest management program. Bull Entomol Soc Am.

[112] Gardner FP, Pearce RB, Mitchell RL. Physiology of crop plants. 1985.

[113] Bonito G, Reynolds H, Robeson MS, Nelson J, Hodkinson BP, Tuskan G (2014). Plant host and soil origin influence fungal and bacterial assemblages in the roots of woody plants. Mol Ecol.

[114] Wagner MR, Lundberg DS, Del Rio TG, Tringe SG, Dangl JL, Mitchell-Olds T (2016). Host genotype and age shape the leaf and root microbiomes of a wild perennial plant. Nat Commun.

[115] Van der Putten WH, Bardgett RD, Bever JD, Bezemer TM, Casper BB, Fukami T (2013). Plant-soil feedbacks: The past, the present and future challenges. J Ecol.

[116] Fierer N, Bradford MA, Jackson RB (2007). Toward an ecological classification of soil bacteria. Ecology.

[117] Freilich MA, Wieters E, Broitman BR, Marquet PA, Navarrete SA (2018). Species co-occurrence networks: Can they reveal trophic and non-trophic interactions in ecological communities?. Ecology.

[118] Wolfert S, Ge L, Verdouw C, Bogaardt MJ (2017). Big data in smart farming – A review. Agric Syst.

[119] Busby PE, Soman C, Wagner MR, Friesen ML, Kremer J, Bennett A (2017). Research priorities for harnessing plant microbiomes in sustainable agriculture. PLoS Biol.

