# Supplementary material for: Agricultural intensification reduces microbial network complexity and the abundance of keystone taxa in roots
Source: ISME J. 2019 Mar 8;13(7):1722–36. doi: 10.1038/s41396-019-0383-2 (PMC6591126; doi:10.1038/s41396-019-0383-2)
Supplement: Supplementary file 1 — Supplementary Information [file 41396_2019_383_MOESM1_ESM.docx]

**Supplementary methods**

*DNA extraction, PCR cycling and SMRT sequencing*

A 20 mg of roots (dry weight) was used for DNA extraction using 600 mL of NucleoSpin lysis buffer PL1 for 15 min at 65 °C followed by the NucleoSpin Plant II kit (Macherey & Nagel, Düren, Germany). The DNA samples were amplified with the primer pair *ITS1F-ITS4* [1, 2] targeting the entire ITS region [3]. The forward and reverse primers were synthesized with a 5-nucleotide-long padding sequence followed by an 8-nt barcode tag at the 5’ end to allow multiplexing of samples within a single sequencing run. PCR was performed using the Phusion High-Fidelity DNA polymerase system (Finnzymes, Espoo, Finland). Reactions were prepared in a volume of 20 μl and triplicate reactions per DNA sample were performed to minimize stochastic PCR effects of individual reactions. Thermal cycling was performed on a Biorad PCR Instrument (Biorad, Hamburg, Germany) with the following conditions: 5 min initial denaturation at 94°C, 30 cycles of 30 s denaturation at 94°C, 30 s annealing at 55°C and 1 min elongation at 72°C and a final elongation of 10 min. The cycle number was kept as low as possible to minimize chimera formation and to be able to interpret sequence abundance in a semi-quantitative manner [4]. Three PCR replicates were performed per sample and amplicons were loaded on a 1% agarose gel to assess PCR efficiency and the lack of PCR amplicons in non-template control reactions. After PCR, replicates were pooled for each sample and the concentration of amplicon DNA was determined using PicoGreen (Molecular Probes Inc., Eugene, Oregon, USA) on the Varian Turbo GTI fluorescence plate reader (Varian Inc., Poalo, CA, USA).

Amplicon libraries (~2 μg) were built by pooling barcoded DNA samples and purifying twice with the Agencourt AMPure XP PCR Purification system (Beckman Coulter, IN, USA). A final volume of 100 μl was obtained by eluting libraries in sterile miliQ water. The hairpin sequencing adapters (SMRTbellTM templates, DNA Template Prep Kit 2.0 (250bp-<3Kb), p/n 001-540-726) were ligated to the purified amplicon libraries following the blunt-end ligation protocol of PacBio (2kb library preparation) at the Functional Genomic Centre Zurich (FGCZ; [http://www.fgcz.ch](http://www.fgcz.ch/) Zurich, Switzerland). Sequencing libraries were purified with AMPure and assessed for quality (Bioanalyzer, Agilent, CA, USA) and quantity (Qubit 1.0, Thermo Fischer Scientific, NY, USA). Libraries were finalized for sequencing by annealing the sequencing primers to the SMRTbells and binding of the DNA polymerase to the template complex. The sequencing libraries were prepared using P6/C4 chemistry (DNA/Polymerase Binding Kit P6 (p/n 100-372-700), DNA Sequencing Reagent 4.0 (p/n 100-356-200)) on the PacBio® RS II Instrument (p/n 100-210-100). All kits were purchased from Pacific Biosciences and used according to the manufacturers’ protocols (Pacific Biosciences, CA, USA).

*Sequence data processing*

The SMRT Portal (v1.4) was used to extract from the raw data the circular consensus sequences (CCS) of at least five passes (CCS reads present the consensus of at least five subreads) in fastq format using default parameters for consensus base calling. The CCS reads were processed in Mothur (v.1.35.0) [5] and we followed the specific recommendations for PacBio data [6]. We converted the data from fastq to fasta and qual formats using the command fastq.info with the option pacbio=T. With the command trim.seqs we selected reads ranging from 532 bp to 1.2 kb in length (minlength=532, maxlength=1200) and filtered for quality sequences consisting of reads that do not contain ambiguous base calls (maxambig=0). The CCS reads contain forward and reverse-complement oriented sequences of the amplicon insert because the blunt-end ligation of the sequencing adaptor to the PCR amplicon is not directional. Hence, we reverse complemented (using the command reverse.seqs in Mothur) and doubled (bash code: cat sequences.fasta sequences.rc.fasta > sequences.doubled.fasta) the quality reads for subsequent demultiplexing. Reverse complementing and doubling of the quality reads permitted to assign the reads in immediate proper orientation to their respective barcodes and samples. Quality reads were demultiplexed based on the barcode-primer sequences allowing a threshold of one mismatch (Schloss et al. 2016) using *flexbar* (Dodt et al. 2012). Flexbar was set to trim the barcode and primer sequences from the quality reads upon demultiplexing. We used a custom BASH code to name each sequence passing quality filtering and demultiplexing with a unique header containing the information to which sample it belongs. This was performed by adding identifiers of both barcodes (e.g. ‘F2’ and ‘R3’) to the headers of the fasta sequences and a unique sequence number (e.g. ‘441’). We clustered the quality sequences into operational taxonomic units (OTUs) at 98% sequence similarity with the Uparse series of scripts (Edgar, 2013). Reads were sorted by abundance, de-replicated and single-count and chimeric sequences were excluded for OTU delineation. Sequences with a global abundance of less than 0.1% and sample-specific abundance of less than 0.5% were removed based with a custom R code. OTUs were classified taxonomically using a QIIME-based wrapper of BLAST [7] against the UNITE database (version 7, 01.08.2015) [8]. The OTU and taxonomy tables were filtered to exclude OTUs classified as non-fungal with a custom R code.

**Table S1.** Indicator taxa in the wheat root microbiota under three farming systems. These taxa were identified from the whole dataset across three farming systems using the *indicspecies* package in R. Only significant taxa were selected for each farming system.

| **Farming Systems** | **Order** | **Family** | **Genus** |
| --- | --- | --- | --- |
| *Conventional* |  |  |  |
| OTU_580* | *Hypocreales* | *Hypocreaceae* | *Trichoderma* |
|  |  |  |  |
| *No-tillage* |  |  |  |
| OTU_380** | *Chaetothyriales* | *Chaetothyriaceae* | *Cyphellophora* |
| OTU_69* | *Sordariomycetes_ord_Incertae_sedis* | *Sordariomycetes_fam_Incertae_sedis* | *Myrmecridium* |
| OTU_261* | *Pleosporales* | *Phaeosphaeriaceae* | *Phaeosphaeria* |
| OTU_510* | *Helotiales* | *Helotiales_fam_Incertae_sedis* | *Cadophora* |
| OTU_448* | *Pleosporales* | *Pleosporales_fam_Incertae_sedis* | *Pyrenochaeta* |
| OTU_816* | *Filobasidiales* | *Piskurozymaceae* | *Solicoccozyma* |
| OTU_313* | *Agaricales* | *Bolbitiaceae* | *Conocybe* |
|  |  |  |  |
| *Organic* |  |  |  |
| OTU_391** | *Sordariales* | *unidentified* | *unidentified* |
| OTU_47* | *Cantharellales* | *Ceratobasidiaceae* | *unidentified* |
| OTU_110* | *unidentified* | *unidentified* | *unidentified* |
| OTU_137* | *Sordariales* | *Chaetomiaceae* | *Chaetomium* |
| OTU_828* | *Agaricales* | *Psathyrellaceae* | *Psathyrella* |
| OTU_456* | *Cantharellales* | *Ceratobasidiaceae* | *unidentified* |

** and * indicate statistical significance at P<0.01 and P<0.05 level

**Table S2.** Keystone taxa in the overall network. Keystones were selected on the basis of high degree, high closeness centrality and low betweenness centrality (cut-off: degree >50 and CC >0.44 and BC <0.12) according to Berry and Widder (2014). Members of arbuscular mycorrhiza are highlighted in bold.

| **OTU-id** | *Order* | *Degree* | *Closeness centrality* |  | *Betweenness centrality* |
| --- | --- | --- | --- | --- | --- |
| OTU_36 | ***Glomerales*** | 83 | 0.502 |  | 0.120 |
| OTU_11 | *Tremellales* | 82 | 0.494 |  | 0.112 |
| OTU_4 | ***Paraglomerales*** | 79 | 0.483 |  | 0.101 |
| OTU_18 | *Tremellales* | 73 | 0.482 |  | 0.053 |
| OTU_10 | *Tremellales* | 68 | 0.473 |  | 0.054 |
| OTU_43 | ***Glomerales*** | 65 | 0.472 |  | 0.051 |
| OTU_42 | ***Glomerales*** | 65 | 0.459 |  | 0.041 |
| OTU_58 | ***Diversisporales*** | 60 | 0.455 |  | 0.065 |
| OTU_160 | ***Glomerales*** | 54 | 0.446 |  | 0.034 |
| OTU_185 | ***Paraglomerales*** | 52 | 0.463 |  | 0.023 |
| OTU_86 | ***Diversisporales*** | 52 | 0.440 |  | 0.031 |
| OTU_76 | *Malasseziales* | 50 | 0.452 |  | 0.027 |
| OTU_23 | *Cantharellales* | 50 | 0.440 |  | 0.060 |

**Table S3.** Keystone taxa in root mycobiome under different farming systems. Keystones were selected on the basis of high degree, high closeness centrality and low betweenness centrality (cut-off: degree >15 and CC >0.28 and BC <0.18) according to Berry and Widder (2014). Members of mycorrhiza are highlighted in bold.

| **OTU-id** | *Order* | *Degree* | *Closeness centrality* |  | *Betweenness centrality* |
| --- | --- | --- | --- | --- | --- |
| *No-tillage* |  |  |  |  |  |
| OTU_43 | ***Glomerales*** | 21 | 0.288 |  | 0.153 |
| OTU_64 | Unidentified | 16 | 0.286 |  | 0.180 |
| *Organic* |  |  |  |  |  |
| OTU_11 | *Tremellales* | 32 | 0.380 |  | 0.103 |
| OTU_42 | ***Glomerales*** | 31 | 0.382 |  | 0.139 |
| OTU_18 | *Tremellales* | 31 | 0.365 |  | 0.071 |
| OTU_10 | *Tremellales* | 31 | 0.382 |  | 0.078 |
| OTU_36 | ***Glomerales*** | 26 | 0.349 |  | 0.048 |
| OTU_31 | Unidentified | 25 | 0.361 |  | 0.076 |
| OTU_160 | ***Glomerales*** | 23 | 0.348 |  | 0.097 |
| OTU_14 | *Capnodiales* | 21 | 0.339 |  | 0.051 |
| OTU_129 | *Hypocreales* | 20 | 0.356 |  | 0.051 |
| OTU_6 | *Hypocreales* | 20 | 0.344 |  | 0.046 |
| OTU_22 | Unidentified | 19 | 0.328 |  | 0.055 |
| OTU_76 | *Malasseziales* | 18 | 0.337 |  | 0.020 |
| OTU_59 | *Sebacinales* | 18 | 0.334 |  | 0.020 |
| OTU_221 | ***Glomerales*** | 18 | 0.329 |  | 0.025 |
| OTU_8 | *Pleosporales* | 17 | 0.339 |  | 0.033 |
| OTU_58 | ***Diversisporales*** | 17 | 0.323 |  | 0.023 |
| OTU_161 | *Cystofilobasidiales* | 16 | 0.329 |  | 0.036 |
| OTU_38 | *Helotiales* | 16 | 0.326 |  | 0.027 |
| OTU_155 | *Cystofilobasidiales* | 16 | 0.339 |  | 0.059 |
| OTU_138 | ***Diversisporales*** | 16 | 0.318 |  | 0.028 |
| OTU_4 | ***Paraglomerales*** | 16 | 0.319 |  | 0.038 |
| OTU_1 | Unidentified | 15 | 0.338 |  | 0.162 |
| OTU_120 | ***Diversisporales*** | 15 | 0.325 |  | 0.022 |
| OTU_19 | *Hypocreales* | 15 | 0.304 |  | 0.031 |
| OTU_167 | ***Glomerales*** | 15 | 0.342 |  | 0.031 |
| OTU_86 | ***Diversisporales*** | 15 | 0.330 |  | 0.045 |
| OTU_185 | ***Paraglomerales*** | 15 | 0.335 |  | 0.027 |

**Table** **S4**: Results of the Kolmogorov-Smirnov test comparing bootstrapped node attributes of root fungal networks under three cropping practice. For each network, node attributes were computed by bootstrapping approach with 10,000 iterations. Kolmogorov-Smirnov test compares the overall shape of the cumulative distribution of two variables where the null hypothesis is that the variables have same distribution patterns.

| **Comparison** | **Degree** | **Betweenness** | **Closeness** |
| --- | --- | --- | --- |
| Conventional vs No-till | 0.030** | 0.050*** | 0.724*** |
| Conventional vs Organic | 0.131*** | 0.107*** | 0.801*** |
| No-till vs Organic | 0.127*** | 0.103*** | 0.801*** |

The values in each box represents *D*, which the maximum difference in the absolute cumulative distribution function.

**, and *** indicate statistical significance at P<0.05, 0.01, and 0.001

**Table S5.** Spearman rank correlations among alpha diversity indices and relevant soil properties.

| **Soil properties** | Species richness | Sheldon evenness | Shannon-Weaver index |
| --- | --- | --- | --- |
| Total P | -0.492** | -0.379* | -0.337* |
| Olsen P | -0.564** | -0.453** | -0.385* |
| pH | 0.368* | 0.480** | 0.471** |
| Bulk density | 0.414* | 0.304* | 0.296* |

* and ** indicate statistical significance at P<0.05 and P<0.01

**Figure S1.** A map of Switzerland showing the location of farmlands in the northeastern (around Zurich) and southwestern (around Changins) regions. Samples were collected from 30 farmlands in each region with 20 farmlands for each of the three farming systems (ten per region).


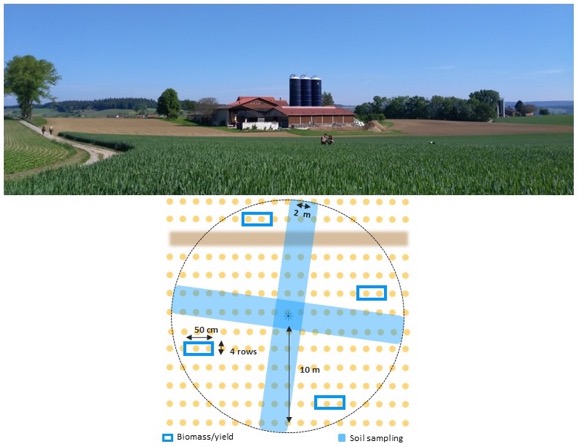


**Figure S2.** A field photo and sampling design in this project. Within each sampling zone, two perpendicular transect lines passing through the centre were defined, which were positioned approximately at a 45° angle to the seedling rows. At each site, ten wheat plants, five per transect, were excavated using a fork spade.


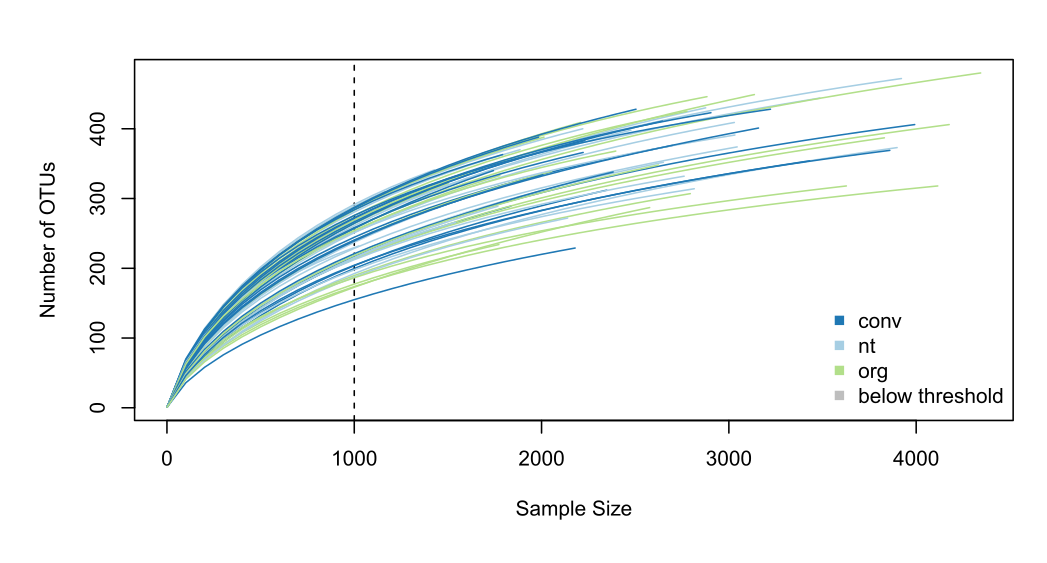


**Figure S3.** Rarefaction curve showing the number of fungal OTUs plotted against number of sequences at 2% sequence similarity. The OTU table was rarefied to 1000 reads per sample.


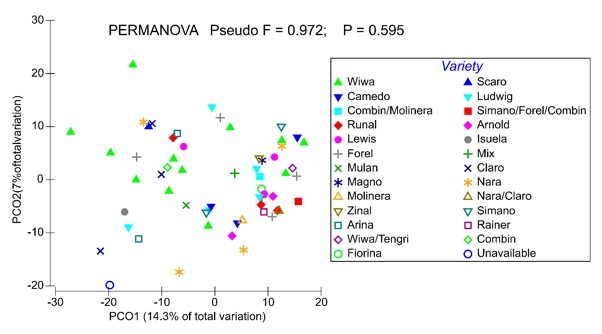


**Figure S4.** Principal coordinate analysis illustrating no significant effect of wheat varieties on the structure of root fungal communities. The results from PERMANOVA further supported it with Pseudo F of 0.959 and a P value of 0.686.


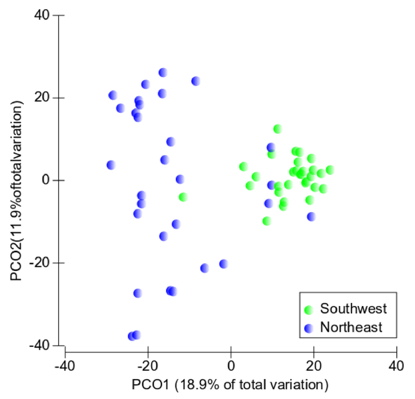


**Figure S5.** Principal coordinate analysis showing the effect (P<0.001) of geographical locations (northeast and southwest regions) on root fungal community structure.


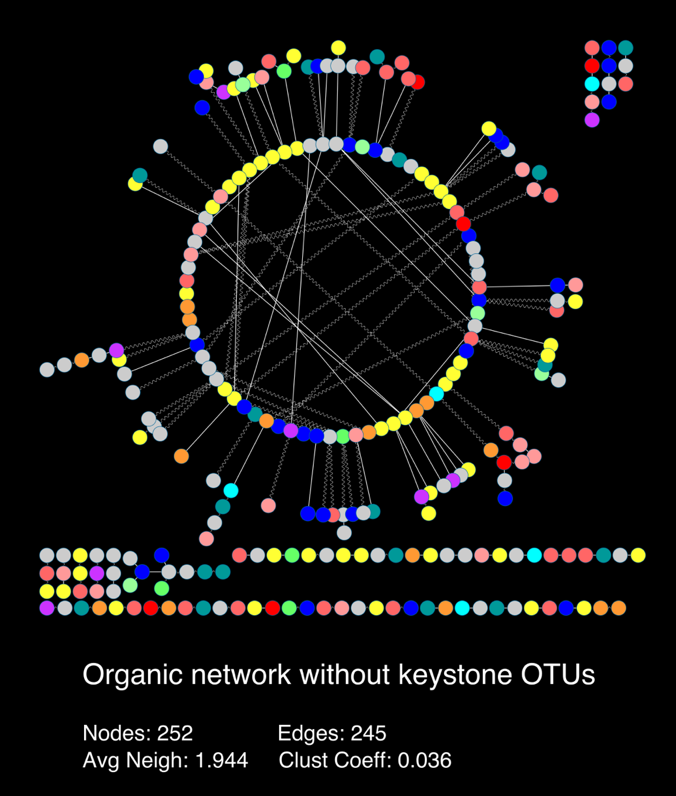


**Figure S6:** Organic network without the keystone OTUs. The network was constructed by removing the 29 OTUs that were identified as keystone taxa under organic farming. Clustering coefficient, average number of neighbours, and the number of nodes and edges were substantially smaller than the original network.

**
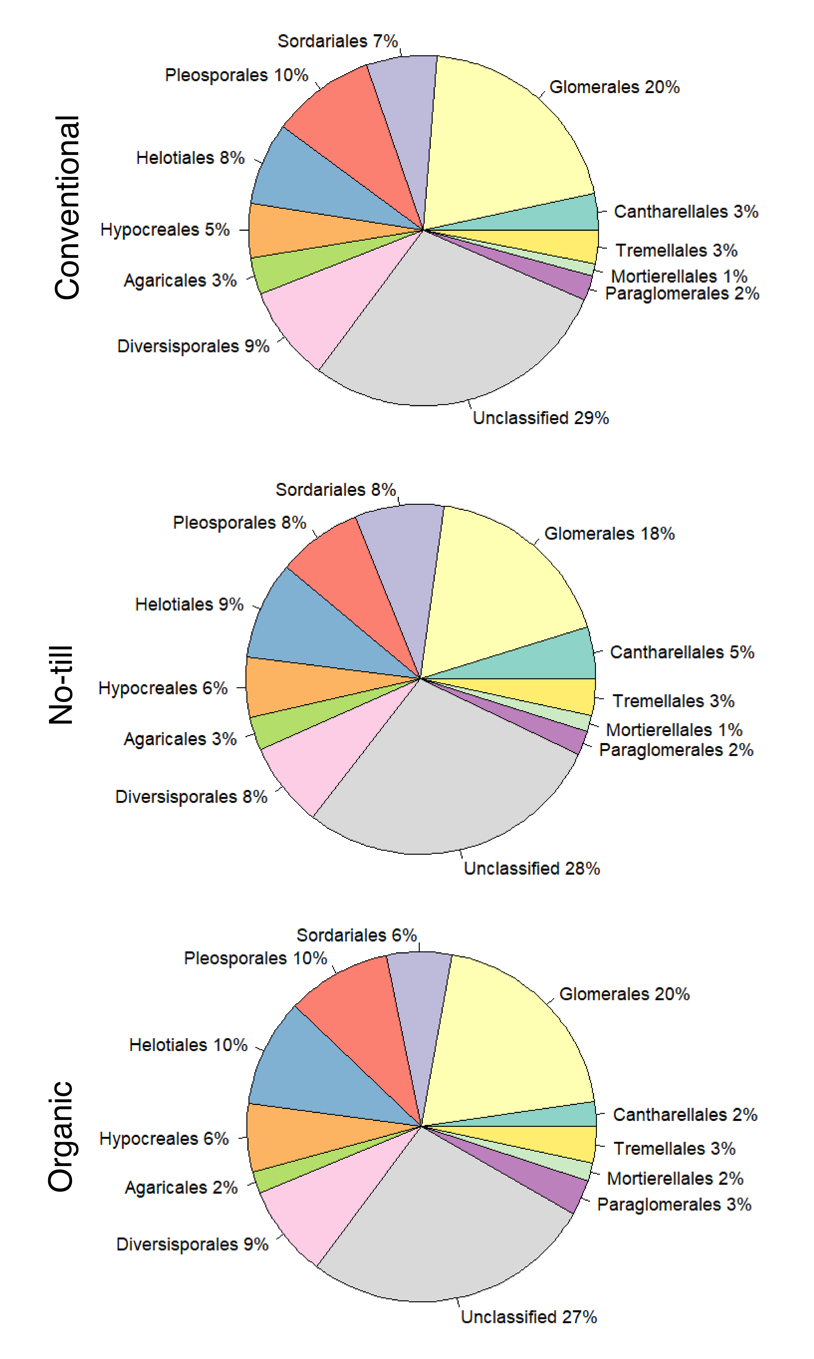
**

**Figure S7.** Node distribution across fungal orders in the wheat root microbiota under three farming systems.


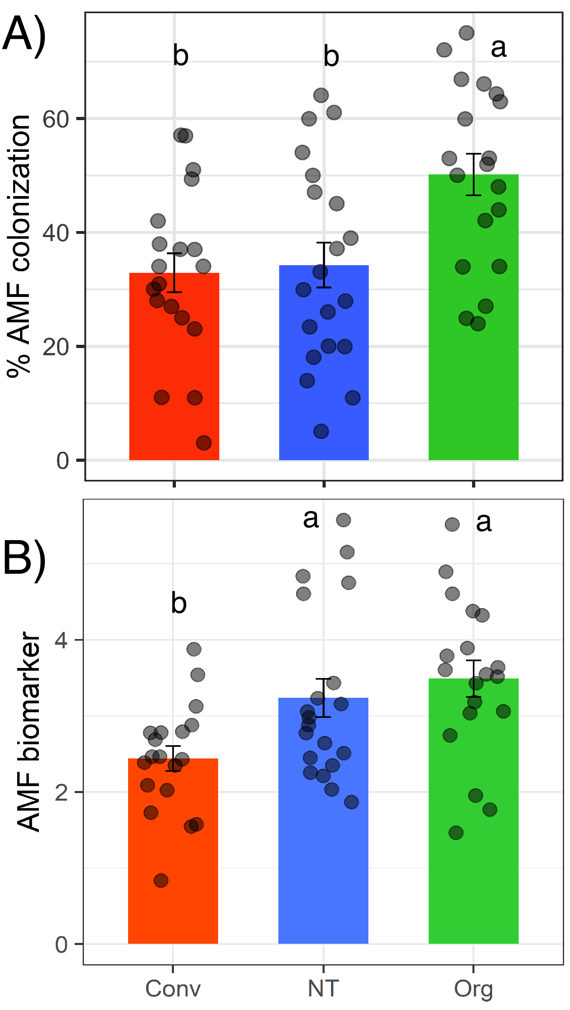


**Figure S8.** A) Root colonization of arbuscular mycorrhizal fungi (AMF) across conventional (Conv), no-till (NT) and organic (Org) farming systems. Percentage of colonization was significantly (P<0.05) higher under organic farming than that of conventional and no-till farming. B) Abundance of AMF in soils as measured by AMF PLFA biomarker 16:1w5. Abundances under organic and no-till farming were significantly P<0.05) higher than that of conventional farming.


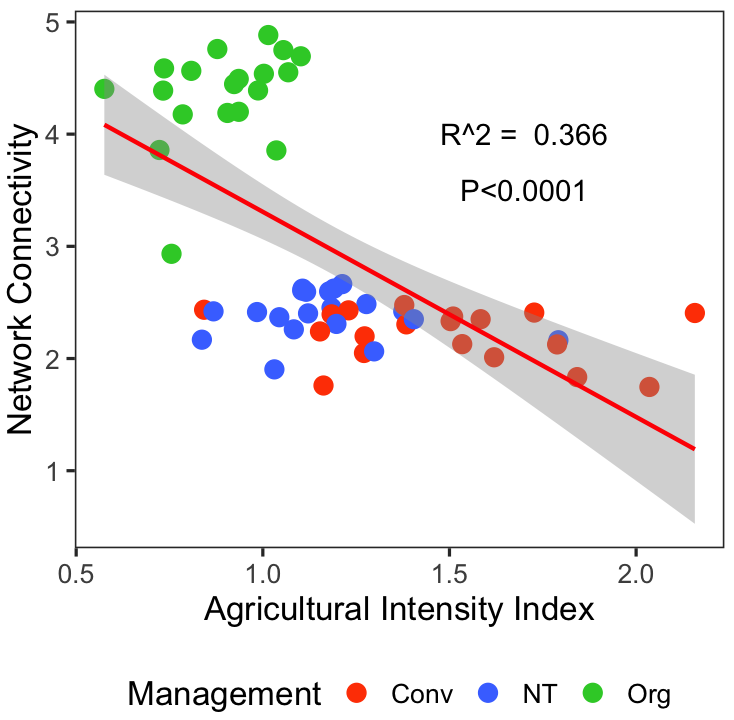


**Figure S9.** Impact of agricultural intensification on network connectivity across conventional (Conv), no-till (NT) and organic (Org) farming systems. Agricultural intensity index was estimated using information on three anthropogenic input factors: fertilizer use, pesticide use and the consumption of fuel for agricultural machineries. Network connectivity is represented by node degrees.

**
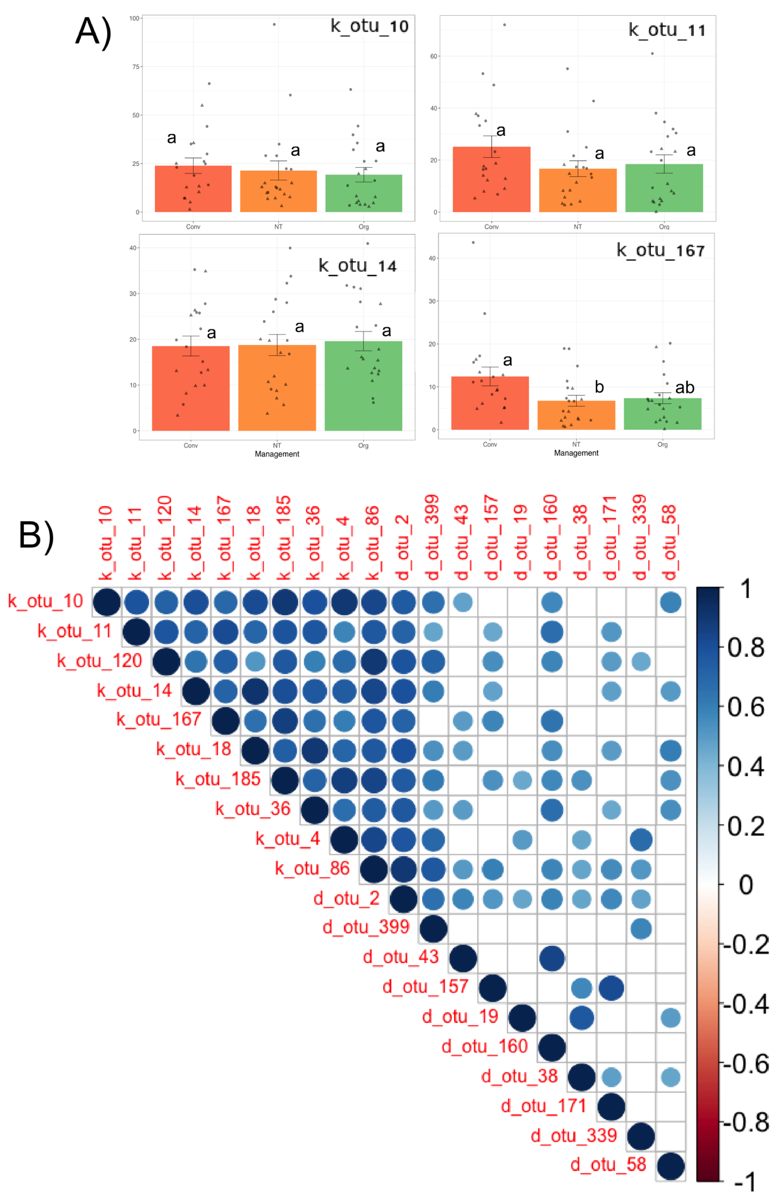
**

**Figure S10.** (A) Barplots showing the sequence abundance of selected keystone taxa (k_otu) across farming systems. Different lowercase letters indicate statistically significant (P<0.05) difference between farming systems. The role of keystone taxa in the microbiota is not determined by their abundance across farming systems. (B) Correlogram showing the significant relationships (P<0.05) between ten keystone taxa (k_otu) and ten dominant taxa (d_otu) under organic farming. Keystone taxa are not only correlated with each other but also with several dominant taxa in the microbiota.

**References**

1. White TJ, Bruns TD, Lee SB, Taylor JW. PCR protocols: a guide to methods and applications. In: Innis MA, Gelfand DH, Sninsky JJ WT (ed). *PCR protocols: a guide to methods and applications.* 1990. Academic Press, New York, pp 315–322.

2. Gardes M, Bruns TD. ITS primers with enhanced specificity for basidiomycetes, application to the identification of mycorrihiza and rusts. *Mol Ecol* 1993; **2**: 113–118.

3. Bodenhausen N, Somerville V, Desiro A, Walser J-C, Borghi L, Heijden M van der, et al. Species-specific root microbiota dynamics in response to plant-available phorphorus. *bioRxiv* 2018; 400119.

4. Lindahl BD, Nilsson RH, Tedersoo L, Abarenkov K, Carlsen T, Kjøller R, et al. Fungal community analysis by high-throughput sequencing of amplified markers--a user’s guide. *New Phytol* 2013; **199**: 288–99.

5. Schloss PD, Westcott SL, Ryabin T, Hall JR, Hartmann M, Hollister EB, et al. Introducing mothur: Open-source, platform-independent, community-supported software for describing and comparing microbial communities. *Appl Environ Microbiol* 2009; **75**: 7537–7541.

6. Schloss PD, Jenior ML, Koumpouras CC, Westcott SL, Highlander SK. Sequencing 16S rRNA gene fragments using the PacBio SMRT DNA sequencing system. *PeerJ* 2016; **4**: e1869.

7. Altschul SF, Gish W, Miller W, Myers EW, Lipman DJ. Basic Local Alignment Search Tool. *J Mol Biol* 1990; **215**: 403–410.

8. Koljalg U, Nilsson RH, Abarenkov K, Tedersoo L, Taylor AFS, Bahram M. Towards a unified paradigm for sequence-based identification of fungi. *Mol Ecol* 2013; **22**: 5271–5277.
